# Stromal-Derived Factor-1 (SDF-1/CXCL12) and Skin Wound Healing Research at the Intersection Between Regenerative Biology and Medicine

**DOI:** 10.3390/ijms27052165

**Published:** 2026-02-25

**Authors:** Rafaela Vaz Sousa Pereira, Mostafa EzEldeen, Ghislain Opdenakker

**Affiliations:** 1 Laboratory of Immunobiology, Department of Microbiology, Immunology and Transplantation, Rega Institute for Medical Research, KU Leuven, B-3000 Leuven, Belgium; rafaelavazsp@gmail.com; 2 Department of Oral Health Sciences, and Paediatric Dentistry and Special Dental Care, University Hospitals Leuven, KU Leuven, B-3000 Leuven, Belgium; mostafa.ezeldeen@kuleuven.be; 3 BIOMAT—Biomaterials Research Group, Department of Oral Health Sciences, KU Leuven & Dentistry, UZ Leuven, University Hospitals Leuven, B-3000 Leuven, Belgium; 4 Royal Academy of Medicine of Belgium, B-1000 Brussels, Belgium

**Keywords:** diabetic foot ulcer, venous leg ulcer, dermatology, pressure ulcer, stromal-derived factor-1, CXCL12, biomaterials, cytokines, growth factors, tissue regeneration

## Abstract

The history of stromal-derived factor-1 (SDF-1), alias CXCL12, started serendipitously and relatively late in the cytokine cDNA cloning era (1975–2000) and evolved at the biological level from progenitor cell-specific chemokine in the bone marrow to multifunctional cytokine with growth factor-like and tissue-regenerative activities. This evolution was parallelled by the integration of SDF-1/CXCL12 within the protein families of chemokines, cytokines and cell growth-promoting recombinant products having the potential for clinical applications. Here, we use this central position of CXCL12 as small signaling protein as an example for future developments in regenerative medicine. We provide context about SDF-1 biology within the field of skin wound healing research and how this compares with studies of other cytokines and growth factors. We also discuss whether SDF-1 formulations may be exemplary for other cytokines used for tissue regeneration. Normal skin wound healing is fraught with delays and complications in patients with specific underlying diseases, such as diabetes, hypertension and other elderly-related comorbidities, skin infections and accidental physical insults. Except for platelet-derived growth factor (PDGF), many cytokines, including vascular endothelial growth factor (VEGF) and epidermal growth factor (EGF), have failed so far in clinical studies of skin wound healing. This is in part due to the fact that (i) the biology of tissue regeneration is complex and insufficiently studied, (ii) in vitro approaches hardly mimic in vivo situations and (iii) commonly used animal models of acute and chronic wounding do not perfectly match human skin wound regeneration. A review of critical cells and molecules in normal skin and their actions in wounded tissue and a balanced comparison of the recent literature are preambles for progress in wound repair. We define advantages and limitations of recent approaches and appeal for more research. In particular, the possibilities of cellular immunomodulation mediated by endogenous and exogenous SDF-1/CXCL12 as a key molecule for skin regeneration are reviewed. Furthermore, biomaterials and scaffolds for the delivery and use of cytokines in precision medicine and aspects of their biofabrication are outlined with SDF-1 as an example. Finally, we indicate how applications of dermatological SDF-1 formulations for skin wound healing may be tailored for applications in other acute and chronic inflammatory conditions and regenerative medicine. Thereby, SDF-1/CXCL12 is placed at the crossroads between recombinant products, cytokines, chemokines and growth factors and occupies a central position between regenerative biology and medicine.

## 1. Introduction

Regenerative medicine aims to mimic natural processes and healing power with the use of man-made artificial means, including cellular and molecular approaches. However, both approaches are still fraught with serious limitations, demanding that regenerative medicine needs more and better research [1]. As of today, the best way to replace dead or failing tissues or organs is by transplantation from human donors. Organ transplantation of kidneys, liver and heart and even lungs and intestinal tissues has moved gradually over about 60 years from experimental to standard treatments [2,3].

Thanks to the more recent progress in stem cell research, specifically to accomplish induced pluripotent stem cells (iPSCs) for therapeutic use [4], cell-based solutions for organ failure have been proposed. However, although optimism drives further developments, many practical issues still need to be resolved while keeping a critical attitude and including sufficient controls in all in vitro experimental, preclinical and clinical research [5]. A side effect of the enthusiasm for stem cell therapy was that purely molecular solutions for regenerative biology and medicine were somewhat pushed into oblivion. One aim of this critical review is to re-activate pharmacological studies in regenerative medicine.

The generation of organs and the regeneration of lost or damaged tissues is intrinsically related to basic questions in developmental biology and immunology. One key question *en vogue* since long ago is how tissue stiffness, e.g., by extracellular matrix (ECM) molecules, influences cell behavior. In recent years, this type of interaction between cells and the local environment, including ECM and its interactions with various immune cells, obtained a boost with discoveries about the molecular substrates of mechanotransduction [6]. Therefore, studies on the effect of tissue stiffness, both endogenous by ECM and exogenous by the use of new biomaterials, may also provide practical applications in regenerative medicine, and an overview of present approaches seems timely. In addition, commonly studied (synthetic) biomaterials possess intrinsic immunomodulating effects through their biophysical characteristics, such as size (nano- or microparticles), surface characteristics (porous, including proteins), polymerization state and solubility (hydrophilic versus lipophilic). The immunomodulating effects of mechanotransduction are briefly outlined in the section on biomaterials. We here evaluate these new trends with the example of SDF-1, already studied under various formulations and together with biomaterials possessing various degrees of stiffness. We aim to rejuvenate cytokine and growth factor research for the next generation of researchers on the basis of an important medical need.

Developmental biology and regenerative medicine studies also converge in answering questions how organs, including skin, bone marrow, spleen and the lymphatic system, are gradually built and whether these processes may be copied in laboratory settings with the ultimate goal of replacing unfunctional cells, tissues and organs with functional ones. Inspired by cell migration studies in embryology [7,8,9] and organogenesis and limb or tail regeneration in amphibians, scientists anticipated the possibilities of regenerative medicine, yet were wondering whether the best approach to tackle regeneration was rather by molecular or by cellular means. As is often the case, the answer lies in between, with both molecular and cellular approaches. Indeed, with the development of molecular methods to generate the above-mentioned iPSCs about 20 years ago, regenerative medicine research has received a boost. iPSCs are generated by dedifferentiation of adult tissue cells by the artificial introduction of four transcription factors, the so called Yamanaka factors OCT4, KLF4, SOX2 and c-Myc. In principle, this is a molecular approach on existing cells from an adult donor and it circumvents ethical issues encountered with the use of human embryonic stem cells [10]. With iPSC technology it became possible to generate organoids in tissue culture for the study of many diseases, e.g., by comparisons of normal and patient-derived organoids and for the study of pharmacological modulators. However, although laboratory-grown tissues are becoming reality, still many limitations, including tumorigenicity, immunogenicity and heterogeneity, remain major obstacles for iPSC applications [11]. In addition, laboratory-grown artificial vascularized organs remain an enigma, and even under the condition that this possibility would become reality, time and accessibility remain obstacles. Natural growth of organs to maturity takes months to years, and accessibility is limited, subject to immunological histocompatibility. Organ shortage and immunogenicity also remain serious limitations in transplantation medicine. To resolve such problems, alternative strategies, including xenotranplantation with organs from genetically engineered pigs, are viewed as approaches for the future, under the condition of further improvements of present experimental studies [12]. Aside from this shortcut to address donor organ shortage, molecular approaches aiming at the use of endogenous mechanisms of tissue and organ regeneration are worthwhile to investigate. Here, an attempt is made to provide insight regarding how SDF-1/CXCL12 may be used as a mediator in tissue repair, with the use of skin wound healing as an example. The CXCR4 receptor of SDF-1/CXCL12 is present on progenitor cells of all three embryonic germ layers (ectoderm, endoderm and mesoderm). This implies that SDF-1/CXCL12, unlike cell-specific growth factors like EGF and VEGF, influences principally all critical cell types for (skin) regeneration and tissue remodeling and is necessary for wound healing and resolution of inflammation.

In summary, whereas transplantation of autologous skin tissue and vascularized skin flaps are used for the treatment of considerable skin burns and in plastic surgery approaches for cancer and trauma, molecular or pharmacological approaches for the enhancement of skin wound healing are insufficiently investigated. Therefore, a need exists to review critical cells and endogenous molecules of normal wound healing with the aim of promoting clinical and mechanism-based research towards optimized pharmacological formulations. We outline commonly used surgical and nursing approaches for various types of skin wounds and compare these with novel possibilities of immunomodulation of skin wound healing, particularly by endogenous and exogenous stromal-derived factor-1 (SDF-1), alias CXCL12.

## 2. The Discovery and Short History of SDF-1/CXCL12

The cDNAs of two isoforms of SDF-1 were serendipitously cloned when mouse bone marrow stromal cells were used to generate an expression library. Mouse SDF-1α and SDF-1β were identified as belonging to a family of small cytokines, all having a CXC-motif at their amino-terminus [13]. Although at the time of this SDF-1 cDNA cloning it was already recognized that SDF-1 belongs to a family of proteins also containing a previously identified member JE, induced by platelet-derived growth factor (PDGF) [14], it was not yet known that SDF-1 might play a role in hematopoiesis, tissue regeneration or chemotaxis. The dawn of the discovery of chemotactic cytokines or chemokines was the identification of granulocyte chemotactic factor-1 protein, alias interleukin-8/IL-8, within the group of Joost Oppenheim at the National Institutes of Health (NIH) and at the Rega Institute [15,16]. The biology of this prototype of chemokine complemented the activities of previously established chemotactic factors, namely C3a and C5a of the complement system, leukotriene B4 and bacterial formyl methionyl peptides. During the years that followed, many additional CXC- and CC-chemokines were discovered, and it was agreed that it was necessary to introduce a new nomenclature for these new chemotactic factors on the basis of cysteine residue conservation and a numbering system based on the history of their cDNA cloning. Thereby, IL-8 and SDF-1 (encoding chemotactic factors with CXC motifs and whose cDNAs were cloned as the 8th and 12th member in this subgroup) were renamed CXCL8 and CXCL12, respectively. It took several more years, and until the dusk of the chemokine discovery era, to functionally characterize SDF-1/CXCL12 produced by bone marrow stromal cells as a chemokine for progenitor cells [17] (see below).

In various aspects, SDF-1/CXCL12 and IL-8/CXCL8 possess different, yet complementary, functions based on the extremely different abundances of circulating human neutrophils (70%) and hematopoietic progenitor cells (less than 1% in newborns and less than 0.05% in adults). Indeed, IL-8/CXCL8 recruits abundant mature neutrophils from the bone marrow to the blood circulation and thereby induces granulocytosis to assist in the drainage of neutrophils towards peripheral sites of inflammation where IL-8 is produced [16,18]. As a complement, SDF-1/CXCL12, constantly produced by stromal cells in the bone marrow, calls the rare and escaped hematopoietic progenitor cells with the surface marker CD34 and the cognate SDF-1 receptor CXCR4 back from blood circulation into the bone marrow. In this way, both IL-8/CXCL8 and SDF-1/CXCL12 are critical factors in regulating leukocytosis and hematopoiesis [18,19]. When it became gradually understood that many different progenitor cells—on the basis of generating all organs and regenerating almost all tissues—all carry CXCR4 for SDF-1 signaling, it also became clear that SDF-1/CXCL12 is a key factor in stem cell biology and regenerative medicine. Detailed information about multiple functions of SDF-1/CXCL12 in normal biology and pathology were reviewed about 5 years ago [20,21]. More recently, the considerably more extensive literature about the biochemistry and the molecular and cellular biology of IL-8/CXCL8 was compared with the more limited information about SDF-1/CXCL12, illustrating further how the functions of inducible IL-8/CXCL8 complement those of homeostatic SDF-1/CXCL12 [22].

Like the research on tumor necrosis factor (TNF), investigations on SDF-1/CXCL12 owe a lot to global investments in cancer and AIDS research. Indeed, SDF-1/CXCL12 research was artificially boosted when it was discovered that its main receptor CXCR4 is a co-receptor for virus entry into lymphocytes [23,24] and is also expressed on cancer cells [25]. TNF, a potent inducer of neutrophil-specific chemokines [22], was once hailed as potential therapeutic for the treatment of cancer. However, TNF inhibitors mainly reached clinical use for the treatment of autoimmune and autoinflammatory diseases, such as rheumatic arthritis and inflammatory bowel diseases, respectively. In analogy with TNF inhibitors, only SDF-1 antagonists are presently used in clinical medicine for mobilization of hematopoietic progenitor cells [reviewed in [20,21,22]] and to treat a rare genetic disease named WHIM syndrome for warts, hypogammaglobulinemia, infections and myelokathexis [26]. However, SDF-1/CXCL12 by itself may have broader applications, certainly if it may be topically applied as in skin diseases. We here review the relevance, importance and potential use of SDF-1/CXCL12 for acute and chronic skin wound healing. Furthermore, studies with the chemokine SDF-1/CXCL12 as an example are used to delineate the advantages and limitations of recent medicinal approaches with hydrogels and other biomaterials as scaffolds for druggable cytokines and growth factors. These insights are instrumental for further developments and improvements of present therapies and may represent regenerative medicine approaches with applications beyond dermatology.

## 3. Why Is It Important to Search for Better Treatments for Skin Wounds?

Although recent studies have provided a wealth of information about the functions of cells and molecules within the skin [27], attempts to turn this knowledge into practical applications for human tissue regeneration and wound healing have generally failed. Nevertheless, the burden of acute and chronic skin wounds is enormous and increasing. The failure of present treatments is partly due to the complexity of skin tissue, limitations of the used in vitro approaches and animal models for translational medicine purposes, and the variety of different types of skin wounds. The increasing demand to find solutions for specific wound entities is linked with increasing population age, yielding age-related comorbidities. Therefore, it is important that ways toward better treatments need to be based on in-depth understandings of normal human skin wound healing and unbiased searches for causal molecular and cellular players, some of which may have beneficial effects in specific healing phases while being detrimental in other phases [28].

**Figure 1:**
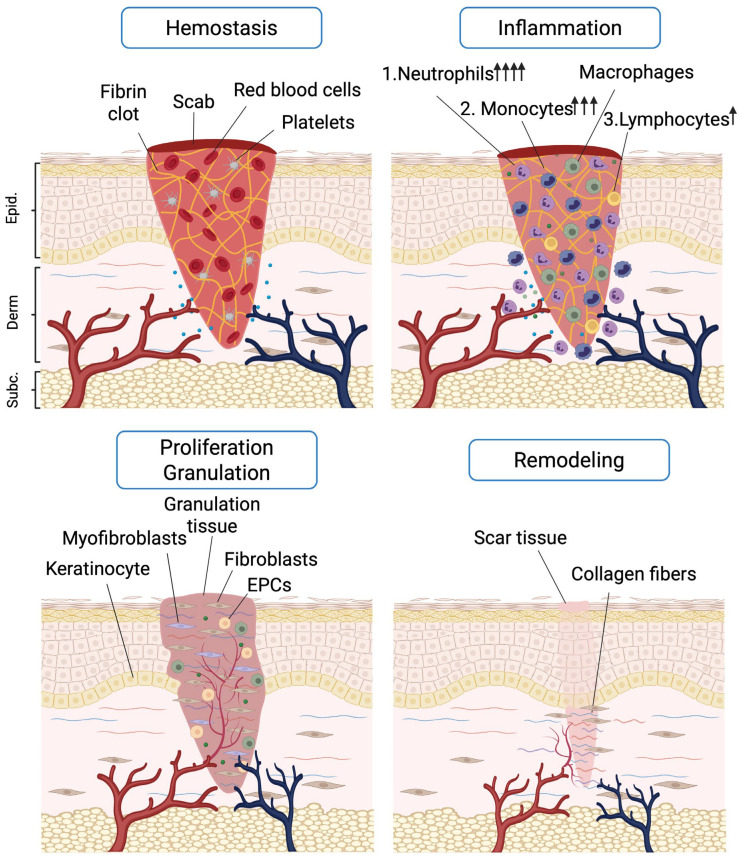
Major cell types associated with the four phases in skin wound healing. The phases are here compared on the basis of major cellular players and differences in time intervals: hemostasis phase within minutes, inflammation within days, granulation or proliferation phase within weeks and remodeling phase taking months to years. The schemes represent sections of three skin layers, named epidermis (Epid.), derm and subcutis (Subc.) and indicated at the left side of the first panel. In the hemostasis phase, bleeding, caused by interruptions in the network of red arterioles or blue venules, is controlled by (i) contraction of pericytes and vascular smooth muscle cells, (ii) coagulant factors and (iii) platelets, leading to the formation of a red blood cell-containing fibrin clot (yellow network) and platelet aggregation and activation with the production of signals for the next phase. In the inflammation phase, immune cells are recruited to injury sites with neutrophils arriving first (1.) and most abundantly (upward pointing arrows) in order to provide protection against microorganisms, initiate new blood and lymph vessel formation and yield signals for recruitment of other leukocytes. Monocytes arrive as second cell type (2.), are less abundant and differentiate into macrophages in the wound. Lymphocytes arrive last. The granulation phase is also named proliferation phase, because new tissue is generated by multiplication of local cell types. Granulation tissue is formed at wound sites, replacing the provisional matrix formed by fibrin. New blood vessels are created by mitosis of local endothelial cells and recruited endothelial progenitor cells (EPCs). Proliferation of myofibroblasts and fibroblasts occurs, and a new extracellular matrix is secreted by these cells that start to contract the wound tissue. This implies that less epithelium is needed to close the wound. Keratinocytes migrate and proliferate from the wound edges and will gradually cover the wound with multiple layers. In the remodeling phase, cellular and matrix debris is removed, extracellular matrix is slowly remodeled and collagen type III fibers are replaced by collagen type I fibers, further contraction occurs, and gradually the epithelial layer is rebuilt by differentiation of keratinocytes from a basal cell layer into the various strata of squamous epithelium. Figure created in BioRender.

Figure 1 illustrates four phases of skin wound healing and major cell players. Details of every phase, including restricted time windows and actions of specific cell types and molecules associated with individual phases, were previously reviewed [27,28]. Such information creates time-dependent opportunities for pharmacological interventions. For the development of molecule-based or pharmacological interventions, it is essential and critical to discriminate whether the presence of cells and molecules is a cause or effect in the healing processes. Indeed, causal relations enable further (pre)clinical research and progress towards therapeutic applications, e.g., for diabetic foot *versus* venous ulcers *versus* pressure sores [28], whereas treatments based on effects are not curative and at best symptomatic. Another concern in the development of novel approaches for skin wound healing is the use of animal models [27]. Whereas genetic knockout studies with e.g., flies, zebrafish and mice, are interesting to define cells and molecules that are essential for wound healing, these species may not provide adequate information about cytokine formulations for use in humans [27,28]. Therefore, preclinical testing needs to be initiated in mammalian hosts, with the mouse as a prioritized compromise. However, despite many impracticalities of large mammalian animals, pigs represent a useful model for skin wound healing with anatomical, histological and some immunological resemblance to human skin. As stated above, because of their physiological and organ size similarities with humans, genetically modified pigs also constitute a preferred pipeline for xenotransplantation purposes into the human species [12]. Indeed, mouse models may be technically easier to perform and less expensive than pig models; however, their skin histology and biology is quite different from that of humans and their circulating and tissue leukocyte numbers differ considerably from those in man and pigs [18].

The skin is a large organ, with the outer layer, the epidermis, composed of keratinocytes, some dispersed melanocytes, and Langerhans cells. Keratin-containing cells provide the human body with a physical shield in two ways: macroscopically by a leather-like barrier and (sub)microscopically by biomolecular barriers in the forms of desmosomes with tight junctions and basement membranes. Melanocytes yield pigmentation protective against UV light, and Langerhans cells, the immune system sentinels of the skin, act as a radar system upon any infectious, chemical, or physical attack. The dermis underneath is mainly produced by fibroblasts and is composed of connective tissue containing extracellular collagen and elastin fibers. These molecules provide further strength to the skin and elasticity between the epidermal shield and underlying tissues. The dermis also houses blood and lymphatic vessels, nerves and their endings, hair follicles, sebaceous and sweat glands, and, aside from fibroblasts and cells of the appendages named above, two other cell types: macrophages and mast cells. The subcutaneous tissue’s deepest layer primarily comprises adipocytes that provide insulation and cushion-like protection for all hard (e.g., bone) and soft (e.g., the liver) organs, as well as energy storage. In summary, the skin is the largest human organ and provides the body with a strong barrier against pathogens, damage from UV light, and mechanical and chemical traumata, and it assists in vital functions such as temperature regulation, sensation, and immunity.

The histological substrates and biochemical components of the skin thus are so complex that they limit simple in vitro research. Nevertheless, technical possibilities increase at a constant pace, in particular for genetic skin diseases. Progress is often made and hope substantiated with exemplary clinical studies, rather than with preclinical animal model studies. An example of this is the replacement of almost the entire human epidermis in a patient suffering from the often lethal disease junctional epidermolysis bullosa. With painstaking and laborious efforts, autologous skin was ex vivo genetically modified to correct the mutation of laminin-332 of a basement membrane component. The gene and stem cell therapy rescued the patient [29]. Another example, highlighting proof-of-principle studies for therapy and the important role of ECM molecules for skin integrity, is the correction type VII collagen with the use of iPSCs in a preclinical mouse model of recessive dystrophic epidermolysis bullosa [30]. Such examples of treatments of rare genetic skin diseases in (pre)clinical settings help to better understand the workings of cells and molecules in normal skin tissue regeneration and raise the hope that more common skin diseases may be adequately treated one day, based on solid experimental evidence.

## 4. Chronic or Hard-to-Heal Wounds

Aside from the burden of acute skin wounds, chronic wounds or hard-to-heal wounds present considerable discomfort for patients and challenges for healthcare professionals. Chronic skin wounds are halted in one of the abovementioned healing phases and often persist for months or even years. With the aging of the population, increasing incidence of diabetes, stroke, and obesity, and persistence of infections, the anticipation is that chronic wounds will remain as a significant clinical, social, and economic burden. The need for improved and new treatments, based on solid biochemical and molecular biological studies, is thus urgent.

Chronic wounds are classified based on their underlying cause, and three major categories are defined: vascular ulcers (venous and arterial), diabetic ulcers, and pressure ulcers [28,31]. Although chronic wounds differ based on these underlying pathologies, they share some common features, which include prolonged or excessive inflammation, persistent infections, biofilm formation, and the inability of dermal and/or epidermal cells to react to healing triggers [32].

Venous leg ulcers (VLUs) account for approximately 70% of all leg ulcers [33]. VLUs develop due to chronic venous insufficiency, a condition where the leg veins have difficulty returning blood to the heart. This is caused mainly by defects/insufficiencies in the venous valves or by venous obstruction, which leads to increased pressure within the veins and causes fluid to leak into the surrounding tissues [34,35]. Over time, the impaired circulation and fluid accumulation cause tissue damage and inflammation and lead to the formation of skin ulcers [34].

Arterial ulcers occur because of reduced blood supply to the lower limbs, resulting in hypoxia and tissue damage [36]. Atherosclerosis and diabetes are major causes of arterial ulcers. Diabetic foot ulcer (DFU) is the most frequently recognized complication of diabetes that affects the lower extremities [37]. The basic mechanism involves the chemical production of so-called advanced glycation end products (AGEs), which occurs when glucose molecules are adducted onto common proteins. By high glucose concentrations in diabetic patients, glucose is chemically reacted by the so-called Maillard reaction, for instance onto collagen or hemoglobin. Glycation needs to be discriminated from glycosylation, which is the enzymatic cellular process of attachment of oligosaccharides to proteins. In diabetes, AGEs act as a danger- or damage-associated molecular pattern (DAMP) and activate inflammatory receptors in small blood vessels and on leukocytes. In the innate immune system, molecular patterns originating from microorganisms, also named pathogen-associated molecular patterns or PAMPs, are recognized by receptors, named Toll-like receptors (TLRs), Rig-like receptors (RLRs) and NOD-like receptors (NLRs) [38]. A number of these receptors also recognize patterns arising when cells die. This led to the concept that infections and cell death alert the immune system by damage or danger signals. Fast post-receptor signaling is critical to direct the slower-acting adaptive immune system into adequate responses, such as antiviral, antibacterial, inflammatory and tissue restorative actions. In diabetes, glycation of collagens as structural components of endothelial basement membranes leads to gradual thickening and thereby impairs transport of oxygen and nutrients. In addition, even low levels of AGEs may interact with their danger/damage receptor, named RAGE, located on neutrophils and endothelial cells and lead to low-grade chronic inflammation. In the long term, these effects result in pathologies in various organs, such as the nervous system, kidneys, and eyes, known as diabetic neuropathy, nephropathy, and retinopathy, respectively. As stated above, glycation occurs on collagens and may indirectly be analyzed by skin autofluorescence as a marker of vascular damage of small blood vessels [39]. The observed thickening of basement membranes of small blood vessels leads to ischemia instigating angiogenesis not only by VEGF but also with the help of SDF-1 [40]. Within the skin, mainly of the lower extremities, this microangiopathy leads to DFUs. The lifetime incidence of foot ulceration in patients with diabetes is a considerable 19–34% [37]. DFUs result in significant morbidity, with high rates of infection requiring hospitalization and even limb amputation. In fact, diabetes is the most common cause of non-traumatic amputations in about 15–20% of DFU patients [37,41].

The etiology of diabetic skin wounds is multifactorial. Diabetic neuropathy involves sensory, motor, and autonomic neural dysfunctions [42,43]. The loss of sensory functions in the extremities reduces the sensation in the feet and leaves injuries and trauma unnoticed. Moreover, motor nerve dysfunction may cause foot deformation, creating pressure points that result in excessive walking friction. Autonomic nerve dysfunction alters sweat production, leaving the foot prone to dryness and skin fissures. Peripheral arterial disease (PAD) is another major factor contributing to the development of DFU [44]. PAD refers to partial or complete occlusion of blood vessels, leading to ischemia and tissue damage. Once a wound appears, many other mechanisms are involved in the impairment of diabetic wound responses. These include persistent inflammatory responses due to overexpression of inflammatory cytokines and dysfunctions in neutrophils and macrophages, impaired angiogenesis due to dysfunctional endothelial cells, decreased levels of angiogenic factors, a reduction in endothelial progenitor cells, and imbalance in extracellular matrix regulation due to high expression of proteases and a decrease in protease inhibitors [45,46,47]. Together, these factors contribute to the progression of DFU.

Pressure ulcers, also known as decubitus ulcers, pressure sores or bedsores, are localized injuries to the skin and underlying tissue developed by long-term pressure between bones and external hard surfaces. These injuries develop mainly over bony prominences such as the sacrum, ischium, and heels [28,48]. Individuals with limited mobility, especially those who are bed- or wheelchair-bound, e.g., because of age-related pathologies or after cerebrovascular accidents or spinal cord injuries, may develop pressure ulcers. Impaired blood flow in the affected area by external pressure leads to tissue damage and necrosis. Other contributing factors include skin moisture and neurological conditions involving the loss of sensory perception or paralysis [49].

Bacterial infection is a common complication contributing to delayed healing in chronic wounds [50]. Bacteria in the chronic wound environment form biofilms, communities of bacteria enclosed in a self-produced matrix, that are present in about 60–80% of chronic wounds and only 6% of acute wounds [51,52]. Chronic wound biofilms contain a diverse polymicrobial community, with *Staphylococcus aureus* and *Pseudomonas aeruginosa* as the most prevalent ones [50]. The biofilm matrix provides bacteria with resistance to antimicrobial treatment and against immune defense by the host [53,54]. Immune responses are often not efficient in eliminating the biofilm-causing pathogen. Instead, inefficient immune responses accelerate surrounding tissue deterioration, assisting in chronic wound persistence [55].

Aberrant wound healing can also lead to the development of pathological scars, such as hypertrophic scars and keloids. Hypertrophic scars are often confined within the boundaries of the original wound and may regress over time, whereas keloids extend beyond the confines of the initial injury site, forming thick and irregular masses that do not regress spontaneously [31,56]. Both conditions are characterized by overproduction and deposition of extracellular matrix (ECM) by fibroblasts. Persistent inflammation is linked with exacerbation of skin fibrosis. The number of immune cells and the levels of inflammatory mediators present at later stages of wound healing correlate with the degree of pathological scar formation [56,57].

## 5. Current Management and Advances in the Treatment of Wounds

The management of chronic wounds has improved in recent years thanks to enhanced prevention, diagnosis, and treatment efforts. However, because of the outlined complexities, wound care is often empirical. Etiology and pathogenesis as outlined above, size and location of wounds, comorbidities, and other host factors are some variables that add complexity to the treatment of chronic wounds. Therefore, each wound should be assessed and treated individually. It is also the hope that with the discovery of new biomarkers and the dissection of causative mechanisms, new pharmacological approaches will improve the burden for many patients [58,59]. We here summarize the present state of skin wound treatment options.

First, causal treatment options need to be considered. For venous ulcers, standard treatment involves compression therapy by applying external pressure to correct the impaired venous blood return [33]. Techniques of compression therapy include bandaging, compression stockings, or intermittent compression pumps used periodically. In the case of arterial ulcers, surgical revascularization of a limb to restore blood supply to the tissues is used, either by clearing out a blockage or with a bypass operation in the legs [60]. To decrease the formation of diabetic ulcers, continuous progress is made in glycemia control by prevention campaigns and new medications. For pressure ulcers, bed-bound or wheelchair-bound patients need repositioning plans. A critical element is keeping the skin clean and dry.

Second, wound bed preparation is a comprehensive approach that focuses on identifying and addressing obstacles to the healing process and promoting the normal healing progress. The so-called TIME framework (**T**issue management, **I**nflammation, and infection control, **M**oisture balance, and **E**pithelial edge advancement) comprises four key principles of wound bed preparation for healthcare professionals to assess and manage different types of wounds [61]. Tissue management involves removing necrotic tissue, debris, or foreign material, and this process is known as *débridement*. This French word stands for the surgical removal of dead or necrotic tissue, often from chronic skin wounds, to restore all elements of the granulation phase of wound healing: exudation, leukocyte infiltration and angiogenesis. It promotes healthy tissue growth and prepares the wound bed for healing. Inflammation and infection control consist of addressing any infection in the wound. Moisture balance involves the control of excessive or insufficient exudate production with the help of dressings. Traditional dressings are dry and intended to cover wounds, whereas modern dressings (foams, films, hydrocolloids, alginate, hydrogels, and nanocomposites) provide appropriate moisture balance, supporting cell growth and formation of new tissue [62,63] (see below). Epithelial edge advancement involves the assessment of the wound contraction and wound re-epithelialization. If no improvement in the healing process is recognized, the elements of the TIME framework need to be re-evaluated and/or advanced therapies considered [32,64].

Advanced therapies may complement standard wound care and are shortly described below. However, conflicting results exist about the efficacy of some of these methods for chronic wound applications. Negative pressure wound therapy involves the application of a negative (sub-atmospheric) pressure to the wound area using a sealed wound dressing connected to a vacuum pump. Hyperbaric oxygen therapy involves placing the patient into a pressurized chamber, exposed to 100% oxygen. Electrical stimulation involves placing electrodes around the wound to deliver short bursts of electrical potential. Frequency, wave amplitude, duration of exposure, and pulse type may be adjusted. Several clinical trials reported accelerated chronic wound healing upon electrical stimulation [65]. Pulsed radio-frequency electromagnetic field is a non-ionizing energy at the shortwave radiofrequency band of the electromagnetic spectrum. A positive outcome in improving wound healing and in reducing wound pain was reported in clinical studies [66]. Extracorporeal shock wave (ESW) therapy involves the delivery of high-energy acoustic pulses to tissues. In clinical practice, three different types of ESW generators are available, equipped with electromagnetic, electrohydraulic, or piezoelectric sources. Experimental studies determined the effect of ESW therapy on cytokines, growth factors, and gene modulation, and clinical evidence suggested beneficial outcomes of this application [67].

Aside from these biophysical advanced therapies, pharmacological approaches have also been tested. Platelet-rich plasma (PRP) refers to a concentration of human platelets in a small volume of plasma obtained by centrifugation of autologous blood samples. This preparation results in a high concentration of growth factors, cytokines and chemokines, primarily derived from platelet α-granules. Clinical studies support the efficacy of PRP for non-healing wounds [68,69]. Becaplermin was the first single cytokine/growth factor preparation for clinical use in wound healing applications. It contains recombinant human platelet-derived growth factor (rhPDGF-BB) in a gel formulation. It was the first commercially available advanced therapy for the treatment of DFUs and remains the only growth factor approved by the Food and Drug Administration (FDA). Becaplermin gel has been shown to promote wound healing in a number of studies [70]. However, Becaplermin treatment is expensive, involves daily application, and thus requires frequent dressing changes. Other pharmacological products containing growth factors for chronic wounds are based on granulocyte–macrophage colony-stimulating factor (GM-CSF), vascular endothelial growth factor (VEGF), epidermal growth factor (EGF), and basic fibroblast growth factor (bFGF) [71,72]. Most of these treatments involve the topical application of recombinant growth factors in the form of solutions, creams, or gels. Generally, repetitive applications are required over a designated period to achieve the desired therapeutic effect. EGF and GM-CSF are injected into the surrounding tissue or directly into the wound bed [72]. In addition, a recombinant human-VEGF (rh-VEGF) gene-carrying plasmid encoding VEGF165 has been tested in patients with diabetic and ischemic wounds. Significant improvement was found in patients treated with this plasmid, although the primary objective of reducing amputation was not achieved [73].

Overall, repetitive administration of proteinaceous growth factors/cytokines is often required due to the hostile environment of chronic wounds, which have high proteinase contents and activities. The proteinase load leads to a rapid loss of bioactivity and degradation of exogenously applied cytokines. Therefore, ongoing research includes the development of sustained-release delivery systems to control and prolong the release of these molecules at the wound site. In this context, gene therapy and a wide range of biomaterials have been investigated as approaches to deliver growth factors during treatment for wound healing [74,75] (see below).

Therapy with stem cells is based on their multipotency and possesses the unique capability of tissue self-renewal. For wound healing applications, the therapeutic potential of stem cells also relies on their capacity to release pro-regenerative cytokines. As stated above, the high expectations for the renewal and organization of all skin cell types into new tissue have not yet been fulfilled. Mesenchymal stem cells (MSCs) from a variety of tissues, such as umbilical cord, bone marrow, skin, and adipose tissue, are used in skin regeneration and wound healing studies and clinical trials [76].

Skin substitutes provide a biocompatible scaffold that supports tissue regeneration, particularly in the case of large surface area wounds [77]. Several commercially available skin substitutes exist, and these can be classified into different categories. Acellular skin substitutes contain components of the ECM, such as collagen, glycosaminoglycans, or decellularized skin molecules. Cellular skin substitutes consist of ECM templates seeded with fibroblasts or with epithelial cells derived from dehydrated human amniotic membranes. Finally, composite skin substitutes consist of both dermal and epidermal components, i.e., a combination of both fibroblasts and keratinocytes (see below).

## 6. Stromal-Derived Factor-1/CXCL12 as an Ideal Agent for Skin Tissue Regeneration in Wounds

As stated above, stromal cell-derived factor-1 (SDF-1) cDNA was cloned [13] before its major chemotactic activity was known [17]. The explanation for this discrepancy is linked to progress of technologies. Chemotactic activity of specific molecules may be demonstrated in vitro by observation of cell migration. To achieve this with the technologies before the year 2000, one needed substantial numbers of cells. For instance, specific human leukocytes were purified into neutrophil, monocyte or lymphocyte populations and used in Boyden chamber chemotaxis tests. Because neutrophils are the most abundant cell type in human circulation, it was straightforward to use these cells first for in vitro leukocyte chemotaxis assays. Thereby, chemokines attracting mainly neutrophils were purified to homogeneity and discovered first, and these contain a CXC motif. Interleukin-8/CXCL8 thereby emerged as the prototype of this neutrophil-specific subfamily [15,16]. The use of mononuclear cells/monocytes in chemotaxis assays enabled the purification and discovery of the first monocyte chemotactic factor, henceforth named MCP-1 and later renamed CC-containing ligand-2 (CCL-2), based on the presence of a CC motif at the amino-terminus [78]. When gradually more CXC- and CC-containing ligands were discovered by protein purification on the basis of chemotactic activities for specific leukocyte types and later also by cDNA cloning experiments a new nomenclature for all chemotactic cytokines (now dubbed chemokines) and their receptors (now subdivided into CXCR and CCR receptor subfamily members) was introduced, carefully reviewed and curated [79,80].

All chemokines signal through specific cell surface G protein-coupled receptors (GPCRs). Chemokines are primarily known to mediate leukocyte recruitment and, thereby, play fundamental roles in immune responses, including the inflammation phase of skin wound healing (Figure 1) [81]. Moreover, chemokines and eventually other chemotactic factors also play key functions in development, homeostasis, and angiogenesis. Within this chemokine receptor-driven paradigm, SDF-1/CXCL12 seems to have a central position, as it acts on precursor cells of both the myeloid (innate immunity) and lymphocytic lineages (adaptive immune system) and because both myeloblasts and lymphoblasts carry CXCR4. However, when myeloblasts and lymphoblasts and lymphocytes differentiate, these cells gradually lose CXCR4 and express specific chemokine receptors for directional migration. During leukocyte differentiation this gradual loss of CXCR4 expression in favor of increases in other chemokine receptors leads to less responsiveness to SDF-1/CXCL12. For lymphocytes, this phenomenon may develop towards a point that SDF-1 even becomes a repellent factor of lymphocytes [82].

**Figure 2:**
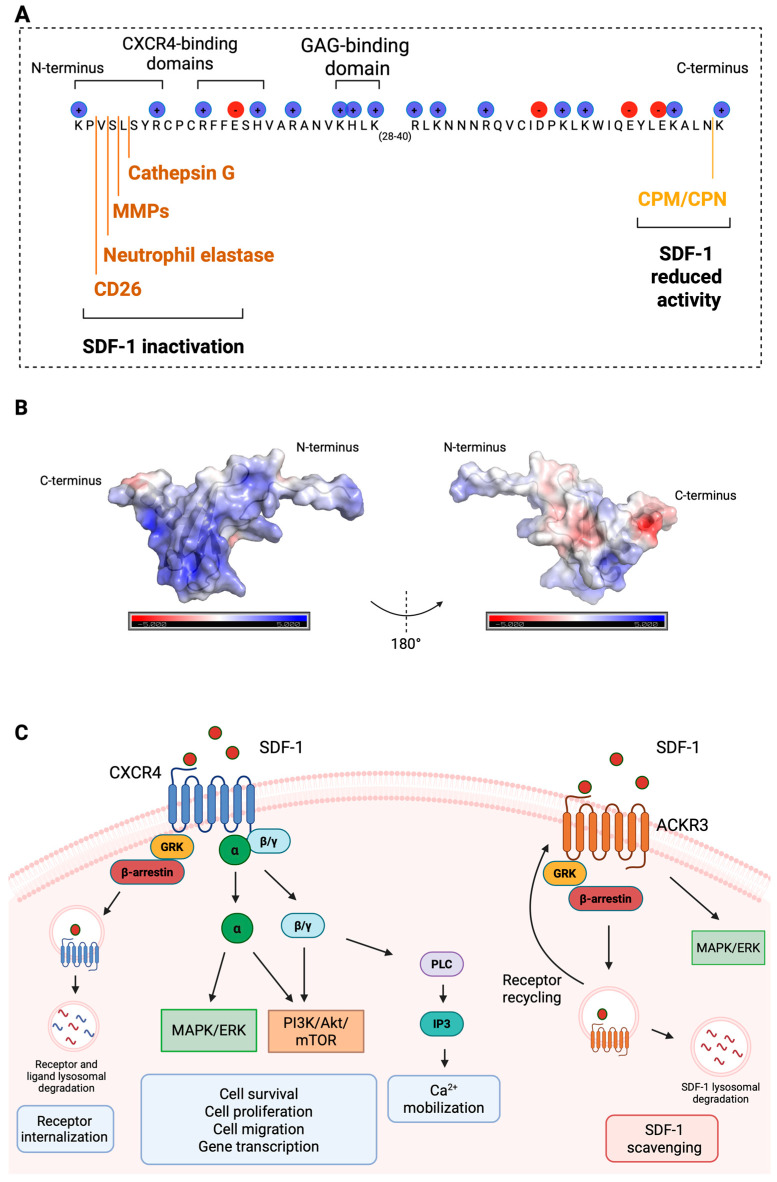
SDF-1 structure and signal transduction. (**A**) Functional parts of the amino acid sequence of human SDF-1α isoform, highlighting positively (blue) and negatively (red) charged amino acids, two CXCR4-binding domains and the GAG-binding domain. Note that amino acid residues from 28 to 40 are not shown here. Several proteases, known to cleave SDF-1 between the indicated amino acids, are indicated in brown color. CD26 is also known as dipeptidyl peptidase IV; CPM and CPN are carboxypeptidase M and N. N-terminal and C-terminal proteolysis inactivate and reduce SDF-1 biological activity, respectively. Illustration modified from [20,22]. (**B**) A 3D surface model of human SDF-1 with indications of the location of positive (blue) and negative (red) charges (PDB structure 2J7Z) [88]. (**C**) Schematic representation of signaling pathways of SDF-1/CXCR4 and between SDF-1 and the atypical chemokine receptor 3 (ACKR3). Activation of CXCR4 by SDF-1 binding induces G protein subunit dissociation and the subsequent activation of PI3K/Akt/mTOR, PLC/IP3, MAPK/ERK pathways and β-arrestin recruitment. Activation of these signaling cascades results in cell survival, proliferation, migration, gene transcription, Ca^2+^ mobilization and receptor internalization. Activation of ACKR3 by SDF-1 binding induces β-arrestin recruitment and ACKR3/SDF-1 internalization. SDF-1 is subsequently degraded in the lysosomal compartment while the receptor recycles to the cell membrane. ACKR3 receptor also results in MAPK signaling. Figure created in BioRender.

In addition to its functional primitive action on both myeloid and lymphoid precursor cells, SDF-1/CXCL12 is also structurally a rather primitive homeostatic chemokine [21] with strong sequence conservation through evolution. Similarly, SDF-1 is implicated in primitive/basic physiological processes linked to stem cells, such as embryogenesis, hematopoiesis, and angiogenesis. Physiological regulation of SDF-1 levels in the bone marrow is under neurological control, and these local levels are subject to circadian oscillations [83]. Deletion of *Cxcl12* or its receptor *Cxcr4* in mice results in a variety of developmental abnormalities that are embryonic-lethal, revealing its essential role in physiology [20,21,22,84,85]. Related to the workings of the immune, vascular and neurological systems in wound healing and tissue repair, it was already early-on recognized that the SDF-1/CXCR4 axis is critical for lympho- and myelopoiesis and for neuron migration [86]. SDF-1 exists in six isoforms in humans (α to φ) and in three isoforms in mice (α, β, and γ) [87]. These forms are differentially expressed across tissues, with SDF-1α being the most expressed and studied isoform (Figure 2). These isoforms arise by alternative splicing (adding extra residues at the carboxyterminus in comparison with SDF-1α) and thereby differ in amino acid sequence. For instance, human SDF-1γ possesses additional residues at the carboxyterminus, resulting in slower proteolytic degradation by carboxypeptidases M and N and possibly thereby longer bioavailability in vivo [20]. Comparative studies of various isoforms are limited and mainly based on in vitro studies with purified, synthetic or recombinant products. Analysis of in vivo functions of individual isoforms is hindered by the fact that (i) all isoforms activate CXCR4 and (ii) discriminative ELISAs to detect all individual isoforms are not yet available [20]. Development of additional analytical tools and isoform-specific genetic knockout or knock-in animals may resolve this information gap.

### 6.1. SDF1/CXCL12 Signaling in a Nutshell

To understand SDF-1/CXCL12 actions, detailed information [20,21] is condensed here related to its molecular structure. Human SDF-1 interacts with the CXC chemokine receptor 4 (CXCR4), expressed on most leukocyte types, hematopoietic progenitor and stem cells, endothelial cells, and various tissue cells. A two-step binding site model has been proposed for the interactions between SDF-1 and CXCR4 [89]. One binding site corresponds to the first eight N-terminal amino acids of SDF-1. The first two amino acids, Lys and Pro, are essential for receptor activation. The other binding site corresponds to the motif RFFESH (residues 12 to 17), which is responsible for the initial contact with CXCR4 and induces conformational changes that promote the interaction of N-terminal amino acids with the receptor and, subsequently, its activation. Proteolytic truncation of N-terminal regions caused by several proteases inhibits CXCR4 activation (Figure 2A).

The binding of SDF-1 to CXCR4 triggers the activation of diverse signaling pathways involved in calcium mobilization, transcriptional activation, receptor internalization, cell proliferation, survival, adhesion, and migration [90,91] (Figure 2C). Upon SDF-1 binding, the receptor undergoes conformational changes that dissociate G protein subunits into the Gα subunit and Gβγ heterodimer, activating downstream effectors. Gα and Gβγ gamma subunits trigger the activation of the PI3K/Akt/mTOR pathway, which is involved in cell survival, proliferation, migration, and gene transcription activation. The Gα subunit activates the MAPK/ERK pathway (Ras/Raf/MEK/ERK). This pathway involves the activation of Ras, a small GTPase, which activates a series of kinases, ultimately leading to the phosphorylation and activation of ERK1/2. The MAPK/ERK pathway activation also regulates cell proliferation, survival, and migration. Moreover, the Gβ/Gγ heterodimer is capable of activating phospholipase C, which generates inositol-(1,4,5)-triphosphate (IP3) and diacylglycerol (DAG). IP3 production results in Ca^2+^ mobilization from the intracellular stores, while DAG promotes the activation of protein kinase C (PKC) and MAPK. Another downstream response involves the phosphorylation of the C-terminus of CXCR4 by G-protein-coupled receptor kinases (GRKs) that induce β-arrestin recruitment. β-arrestin prevents the coupling of CXCR4 with G protein and induces receptor internalization. Additionally, β-arrestin enhances CXCR4-mediated activation of ERK and p38. The JAK/STAT activation pathway may also involve the CXCR4 activation cascade through a G-protein-independent mechanism [92].

In addition to its well-known chemokine receptor CXCR4, SDF-1 also binds to the atypical chemokine receptor 3 (ACKR3), also known as CXC chemokine receptor 7 (CXCR7) [93,94]. ACKR3 primarily functions as a scavenger receptor. By inducing β-arrestin recruitment, ACKR3 is involved in internalization and sequestration of SDF-1 from the extracellular environment. This action helps to regulate the local concentration of SDF-1 and modulate its effects on cells that express CXCR4. Activation of ACKR3 by SDF-1 also results in MAPK signaling. Moreover, ACKR3 can form heterodimers with CXCR4 on the cell surface and mediate cellular responses [95,96].

### 6.2. Interaction of SDF-1/CXCL12 with Polysaccharides and Derivatives

Like many other chemokines, SDF-1 behaves like sugar-binding lectins and interacts with glycosaminoglycans (GAGs). GAGs are long and linear polysaccharide chains consisting of repeating disaccharide units. GAGs are present in the ECM and on the surfaces of cells. GAGs are important mediators of chemokine-induced leukocyte recruitment in vivo through their capacity to bind and immobilize chemokines within ECM and on endothelial cells, thereby forming chemokine gradients required for leukocyte recruitment. GAGs vary greatly in disaccharide structure, linkage, and patterns of acetylation and N- and O-sulphation. They are categorized into six distinct groups: heparan sulfate (HS and heparin), chondroitin sulfate (CS), dermatan sulfate (DS), keratan sulfate (KS), and hyaluronic acid (HA) [97]. SDF-1 contains a GAG-binding domain consisting of a cluster of positively charged amino acids, the BBXB domain, where B stands for any basic (positively charged) amino acid and X for any other residue [98]. This domain consists of Lys, His, Leu, and Lys in the first β-strand of SDF-1 (Figure 2A). In addition to the GAG-binding domain, other positively charged amino acids are also involved in SDF-1/GAGs interaction [20]. The 3D structural arrangement of these positively charged amino acids in the SDF-1 molecule is important for the ability to bind GAGs carrying repetitive negative charges [97] (Figure 2B).

Chlorite-oxidized oxyamylose or COAM is a polysaccharide derivative with repetitive negative charges, thus capable of binding SDF-1/CXCL12, based on mechanisms similar to GAGs. COAM is the final product of a two-step oxidation process starting from the polysaccharide amylose. Amylose is an abundant (and inexpensive) natural polysaccharide made of D-glucose units, bonded to each other through α(1,4) glycosidic bonds. COAM was originally synthesized with the aim of developing an antiviral compound that possesses specific characteristics: polyanionic nature for the induction of interferons, biodegradable backbone, and slow degradation in tissues. More than half a century after its synthesis and description as an antiviral agent, COAM remains a parent polymeric biomaterial born on the basis of and related to cytokine research [99]. The broad-spectrum antiviral effect of COAM was demonstrated in animal models for different virus infections [100,101,102,103]. Although COAM was originally regarded as an inducer of interferon (IFN), later it was demonstrated that COAM works by interaction with chemokines [104,105]. Similar to GAGs, specific chemokines bind to COAM, with kinetics similar to or even faster than those for heparan sulphate, a prototypic GAG [104]. Additionally, COAM induces the expression of specific chemokines in vitro. Local parenteral application of COAM induces considerable leukocyte recruitment in different animal models [104,105,106]. Because COAM does not exhibit chemotactic activity by itself, its indirect effects on leukocyte recruitment are mainly attributed to its binding to endogenous chemokines or induction of chemokine expression [104,107]. The GAG-mimetic features establish COAM as an interesting immunomodulatory compound to study leukocyte recruitment in vivo. In addition, the antiproteolytic effects of COAM were recently described to be beneficial in prolonging the bioactivity of SDF-1 in skin wound healing experiments [108]. With its description in the middle of the 1970s, COAM historically became a parent polymeric biomaterial with antiviral and immunomodulating effects that prove beneficial for skin wound healing and are mediated, amongst others, by the chemokine system. Because of its polymeric nature, hydrophilicity, degradability, high safety index and inexpensive synthesis from abundantly present amylose and its resemblance with GAGs in binding SDF-1, COAM is promoted from a forgotten antiviral agent to an interesting pharmacological agent. As a conclusion, the natural functions of GAGs in the biology of SDF-1/CXCL12, which are mainly based on their repetitive negative charges, may be (bio)chemically reproduced with polymers, such as COAM. Thanks to presently known biological effects of COAM in vivo, this pharmacological agent needs further studies and eventually development into a key reagent for applications in wound healing and regenerative medicine.

### 6.3. Proteolysis of SDF-1/CXCL12

Like many other cytokines, SDF-1 is susceptible to proteolytic truncation by several proteinases, such as dipeptidyl peptidase IV (DPPIV) or CD26, neutrophil elastase, matrix metalloproteinases (MMPs) and cathepsin G [20,108]. These proteases cleave SDF-1 in its N-terminal region, thereby abolishing SDF-1 biological activity (Figure 2A). CD26 exists as a soluble molecule or a cell surface glycoprotein expressed on multiple cells, including leukocytes, fibroblasts, epithelial and endothelial cells. CD26 is a serine protease that cleaves amino-terminal dipeptides with a proline or alanine residue in the penultimate position. In addition to SDF-1, several other chemokines have been identified as CD26 substrates [109]. Neutrophil elastase and cathepsin G are present in the azurophil granules of neutrophils. These serine proteases may act as intracellular antimicrobial agents and are secreted following neutrophil exposure to inflammatory stimuli [110]. In the extracellular milieu, these proteases have a broad—often overlapping—range of substrates. In the proteolysis of SDF-1/CXCL12, neutrophil elastase cleaves behind a valine residue (between aminoterminal positions 3–4 of human SDF-1α), whereas cathepsin G cleaves between positions 5–6 (Figure 2A). MMPs are a family of zinc-dependent endopeptidases consisting of soluble and membrane-bound proteinases, originally described as cleaving ECM molecules [111]. However, the list of substrates of MMPs goes beyond the structural components of the ECM. Substrates of MMPs include growth factors, receptors, adhesion molecules, cytokines, chemokines, and other MMPs [112]. MMP-1, -2, -3, -9, -13 and -14 cleave human SDF-1 between the 4th (Serine, Ser, S) and 5th (Leucine, Leu, L) residues [113]. In contrast to the above-mentioned proteinases, carboxypeptidases (soluble carboxypeptidase N (CPN) and membrane-bound carboxypeptidase M (CPM)) cleave SDF-1 at the C-terminus by removing a lysine residue. This cleavage results in reduced SDF-1 activity (Figure 2A).

As a conclusion, proteolysis of SDF-1/CXCL12 leads to loss of chemotactic activity, a process that may be reversed into enhanced bioavailability with the use of COAM possessing broad-spectrum proteinase inhibitory activity [108].

### 6.4. SDF-1/CXCL12 in Tissue Repair

SDF-1 plays an important role during tissue repair. SDF-1 is upregulated in sites of hypoxia and mediates the recruitment of endothelial progenitor cells (EPCs) to aid in vascular repair [114,115]. In diabetic wounds in mice, reduced expression of SDF-1 has been associated with reduced EPC homing to wound tissue and, consequentially, reduced vascular repair [116,117]. In diabetic patients, a reduction in circulating and tissue SDF-1 has also been observed [118]. Additionally, SDF-1 inhibition was shown to impair even further the healing of diabetic wounds [119]. Besides the described effects of SDF-1 on EPC, SDF-1 also increases the number of transforming growth factor-β- (TGF-β)-producing macrophages in skin wounds [120]. These findings place SDF-1 as a major compound for therapeutic investigations in wound repair. Indeed, several approaches to deliver SDF-1 have been investigated, and these show beneficial outcomes for skin wound healing. These approaches involve daily injections of SDF-1, SDF-1 delivery through liposomes or biomaterials, and on-site SDF-1 production by transformed lactic acid bacteria, by lentiviral vectors or by injected mesenchymal stem cells expressing SDF-1 [116,120,121,122,123,124,125,126,127]. However, an important point to consider involves the fast inactivation of SDF-1 by several proteases (Figure 2A). Therefore, the combination of exogenous SDF-1 with proteinase inhibitors seems necessary for better bio-availability. As described above, COAM mimics the physicochemical properties of natural GAGs and binds to and protects SDF-1 against proteolytic inactivation. The combination of (recombinant) SDF-1 with COAM in a physiological hydrogel brings together three pharmacological principles for wound healing (topical application, protection against proteolysis and prolonged bioavailability) and stimulates further preclinical research even beyond dermatology applications [107]. As a conclusion, SDF-1/CXCR4 has potential for clinical use in skin wound healing and tissue repair applications.

## 7. Biomaterials and Scaffolds for the Delivery of Cytokines in Precision Medicine Applications for Skin Wound Healing

A key element of the complexity of skin wound healing is the gradual replacement of the fibrin network into scar tissue (Figure 1) according to four phases, each involving specific cell types and molecular players (Table 1) [128,129,130,131,132,133,134,135,136,137,138,139,140,141,142,143,144,145,146,147,148,149,150]. This aspect is under intense investigation, with studies about biomaterials providing a supportive framework that mimics ECM and promotes cell adhesion, proliferation and differentiation [151]. Human ECM may be seen as a protective semisolid tissue element composed of macromolecules, mostly collagens and GAGs, produced by connective tissue fibroblasts in normal tissues. Epithelial, mesothelial and endothelial cells may produce basement membranes as ECM. In acute wounds, a provisional ECM is produced by the host under the form of a fibrin clot (Figure 1, Hemostasis phase), whereas in chronic skin wounds bacteria may generate a biofilm as their own protective ECM.

The use of biomaterials and scaffolds in delivering cytokines and growth factors offers many advantages. Firstly, they enable the controlled and sustained release of therapeutic agents directly at wound sites, which is critical for maintaining effective local concentrations over extended periods. This localized delivery may reduce systemic side-effects and improve the overall efficacy of the treatment. Secondly, biomaterials and scaffolds can be engineered to possess specific physical, e.g., stiffness (vide supra), and chemical properties beneficial to wound healing. For instance, their porosity and degradation rate can be tailored to match the healing process, from providing structural support during the initial repair phase to gradual degradation as the new tissue forms. This dynamic interaction may support the natural healing process and promotes tissue regeneration [151,152]. Moreover, integrating growth factors and cytokines into biomaterials and scaffolds may directly influence the inflammatory phase of wound healing. The activity modulation of immune cells, such as neutrophils, macrophages, and T cells, may thwart away infection, accelerate the transition to the proliferative healing phase, and help resolve chronic inflammation [152].

Polymers are the primary biomaterials used in delivering immunomodulators for skin wound healing and can be broadly categorized into natural (biopolymers), synthetic, or hybrids of the two [152,153]. Natural polymers and derivatives, such as COAM, are derived from biological sources and are known for their degradability, biocompatibility, and ability to promote cellular functions, whereas synthetic biomaterials are engineered to possess specific properties such as controlled degradation rates and mechanical strength [151,153]. In the following subsections, we review major properties of the most commonly used natural and synthetic polymers in delivering skin wound immunomodulators, many of which have already been used in conjunction with SDF-1. Our review also includes biomaterials that have not yet been tested as carriers of SDF-1/CXCL12; these are named in order to stimulate such doping studies with SDF-1/CXCL12 and other cytokines. As polymeric biomaterials are often used as co-polymers to overcome their disadvantages or enhance performance, within the next section we will also discuss examples of their application in skin wound healing and on the design and biofabrication of scaffolds.

### 7.1. Collagen and Derivatives

Collagen as an endogenous natural structural protein of the ECM plays a significant role in wound healing. Exogenous collagen preparations are also therapeutically used as biomaterial with the advantages of biocompatibility, low immune response, and low cytotoxicity. Collagen naturally promotes cell adhesion, migration and growth because of its structural features [154]. An advantage of collagen is high tensile strength, and therefore, it can be processed into various forms, such as sponges, hydrogels, and sheets. Some disadvantages of collagen are the lower physical strength, rapid degradation, and contraction [152,155]. Gelatin is a semi-solid/soluble protein preparation derived from collagen by hydrolysis, which breaks the natural triple-helix structure of collagen into (partially) single-stranded molecules. Gelatin is less immunogenic than collagen and retains informational signals such as the arginine–glycine–aspartic acid (RGD) sequence, promoting cell adhesion, migration, proliferation, and differentiation [156]. The mechanical and physical properties or degradation rates of collagen and gelatin can be customized by selecting crosslinkers or forming hybrid scaffolds [155].

### 7.2. Chitosan

Chitosan is a natural polysaccharide obtained by the partial deacetylation of insoluble chitin. Chitin is a copolymer of N-acetylglucosamine and glucosamine residues linked by β(1-4)-glycosidic bonds. It is a natural polymer and structural element in the exoskeleton of crustaceans (such as crabs and shrimps), fungi and insects [157,158]. Chitosan has attracted much attention for skin wound healing applications mainly because of its intrinsic antimicrobial [159] and immunomodulatory properties [160,161], in addition to biocompatibility, biodegradability, low immunogenicity, and gel-forming ability [162]. Moreover, it can be processed in different forms, such as membranes, sponges, hydrogels, nanofibrous scaffolds, and micro/nanoparticles [153]. Chitosan’s drawbacks are low strength, difficulty of controlling pore size, possible toxicity caused by chemical modifications, and the fact that it is mainly soluble in an acidic environment [158].

### 7.3. Hyaluronic Acid

Hyaluronic acid is a glycosaminoglycan present in the ECM of connective tissue and is a significant component of the skin (vide supra). It has the benefits of biocompatibility and low immunogenic potential [163]. Moreover, hyaluronic acid is enzymatically degraded by hyaluronidases to nontoxic by-products easily removed by the body [162]. Hyaluronic acid is highly water-soluble and possesses poor mechanical properties and a rapid degradation rate in vivo. Such disadvantages can be improved by crosslinking and chemical modification [164]. For example, crosslinked hyaluronic acid can form a stable 3D scaffold containing SDF-1/CXCL12 to capture migrating endogenous stem cells for wound healing [165].

### 7.4. Fibrin

Fibrin is formed by the catalytic conversion of fibrinogen by thrombin in the presence of Ca^2+^. Fibrin is typically used as hydrogel, and it has been extensively studied as a biomaterial for different tissue engineering and regenerative medicine (TERM) and clinical applications [166,167,168]. The advantages of this biopolymer are a low cost, good biocompatibility, limited immunogenicity, good reproducibility, excellent cell adhesion properties [169], and the property of stimulating angiogenesis [170]. After enzymatic conversion of fibrinogen into fibrin monomers by the protease thrombin, the monomers undergo self-assembly and lateral aggregation to form protofibrils packed into fibers, forming branched fibrous networks [171]. Factor XIIIa promotes the formation of covalent bonds between fibrin(ogen) peptides to form a mesh network of fibrin fibers [166]. The fibrous network and mechanical properties of fibrin can be tuned by altering the composition [172]. For instance, higher concentrations of factor XIIIa increase fibrin stiffness by catalyzing fibrin covalent crosslinking and compacting fibers [173].

### 7.5. Polyethylene Glycol

Polyethylene glycol (PEG) is a biocompatible synthetic polymer with low toxicity and is degradable in vivo [154]. The main disadvantage is the minimal or absent biological activity due to the non-adhesive nature of PEG chains [174]. PEG hydrogels modified with RGD peptides promote cell adhesion, cell survival and matrix formation within the 3D scaffold [175]. PEG hydrogel’s mechanical strength depends on the molecular weight, crosslinking, and concentration of polymers [174]. Adding polylactic acid (PLA) groups can make it a suitable hydrogel for the controlled release of bioactive molecules [155,176].

### 7.6. Polyvinyl Alcohol

Polyvinyl alcohol (PVA) creates hydrophilic scaffolds with good mechanical properties and biocompatibility. PVA hydrogels are often combined with other polymers to enhance their properties and functionalities [177]. PVA-based materials provide a hydrated environment conducive to wound healing. They support cell proliferation and can be designed to release therapeutic agents over time, improving overall wound-healing outcomes. The high water content and flexibility of PVA hydrogels make them suitable for various wound types [176,177].

### 7.7. Polycaprolactone, Poly-Lactic Acid, and Poly-Glycolic Acid

Polycaprolactone (PCL), Poly-lactic acid (PLA), and Poly-lactic-co-glycolic acid (PLGA) are biodegradable polyesters with a slower and tunable degradation rate, making them suitable for long-term applications in TERM [178]. These FDA-approved biomaterials can be fabricated into various forms, such as nanofibrous membranes, hydrogels, and composite scaffolds, using electrospinning and 3D printing [177,179,180]. This versatility allows for the creation of structures that mimic the ECM of the skin, providing physical support and an ideal environment for cell growth and tissue regeneration. The scaffolds can be engineered to release anti-inflammatory agents, growth factors, and other immunomodulators in a controlled manner. Specifically, they have been used to deliver agents such as hydrogen molecules and the chemokines IL-8/CXCL8 [181] and SDF-1/CXCL12 [182].

This polyester family is often combined with other polymers and bioactive molecules to enhance the mechanical properties and biological performance. For example, blending PLGA with natural polymers like collagen and chitosan can improve its biocompatibility and degradation profile [177,183].

In conclusion, various polymeric substances, including primary and chemically modified natural polymers and synthetic compounds, may assist in enhancing the activities of cytokines and growth factors in skin wound healing. Hydrogel formulations have the advantage of doping with cytokines or chemokines and proteinase inhibitors while still being injectable and yielding localized and gradual release of active substances. The combination of such scaffolds with cytokines for therapeutic applications needs careful (pre)clinical studies, because many scaffolds by themselves affect cell behavior, possibly by their stiffness through mechanotransduction effects and by alterations in cytokine and growth factor production.

## 8. Biofabrication of Scaffolds for the Delivery of SDF-1/CXCL12

The design and structure of the scaffold are deterministic for cell attachment and proliferation and subsequent wound healing, as illustrated by the examples in the previous section. Because scaffolds in combination with cytokines and growth factors are medically applied at an increasing pace, basic elements of their biofabrication are covered here. Important design considerations include the architecture of the porosity and interconnectivity, hydrophobicity, hydrophilicity, and stiffness [155,176]. Scaffolds used to deliver immunomodulators for wound healing are usually in injectable hydrogels, sponges, sheets, or are biofabricated using electrospinning and 3D printing [177,178,182,184,185]. Table 2 summarizes the methods of immunomodulator incorporation, controlled release mechanisms, and the method of testing bioactivity. As already mentioned in the Introduction section, it needs to be added here that alterations in the stiffness of ECM and, by extrapolation, of any (bio-)fabricated scaffold, will change mechano-transduction signals, also interacting with the immune system. As an example, integrins, being important mediators of cell adhesion in the immune system, also act as sensors of mechano-signals [186].

Liu and colleagues developed a gelatin-based hydrogel microcarrier system functionalized with stromal cell-derived factor 1 (SDF-1/CXCL12) and coated with Matrigel (MC-SDF-1-Mat) to enhance skin flap repair. The microcarriers were prepared by loading SDF-1 onto gelatin-based microcarriers through a freeze-drying process. Matrigel, a collagen protein mixture, was used to coat the SDF-1-loaded microcarriers. This coating was applied through multiple freeze-drying steps to create a sustained release system for SDF-1. The coated microcarriers exhibited a porous surface, enhancing the sustained release of SDF-1. This was confirmed by drug release experiments showing significantly prolonged release profiles compared to uncoated carriers. The MC-SDF-1-Mat microcarriers significantly promoted human umbilical vein endothelial cells (HUVECs) proliferation, migration, and angiogenesis. In a murine random skin flap model, the MC-SDF-1-Mat-treated group significantly reduced the flap necrosis area. Immunohistochemical analysis revealed the highest degree of neovascularization in the MC-SDF-1-Mat group, demonstrating the effectiveness of SDF-1 in promoting angiogenesis and tissue repair [185]. In another study, an injectable hydrogel scaffold composed of catechol-modified oxidized hyaluronic acid (OD), methacrylated gelatin (GM), and quaternized chitosan (QCS) was loaded with adipose mesenchymal stem cell-derived exosomes (Exos). The hydrogel was cross-linked using visible light, providing injectability and biocompatibility suitable for in situ gelation. The OD/GM/QCS-Exo hydrogel exhibited significant immunomodulatory properties, primarily due to the sustained release of exosomes. The exosomes activated immune regulation by reducing inflammatory responses, as evidenced by decreased expression of pro-inflammatory cytokines TNF-α and IL-6. This reduction in inflammation helps to create a conducive environment for effective wound healing and tissue regeneration [184].

Augustine et al. developed a co-electrospun hydrophilic/hydrophobic bicomponent membrane scaffold. The scaffold comprises polyvinyl alcohol (PVA) for the hydrophilic component and polycaprolactone (PCL) for the hydrophobic component. SDF-1/CXCL12 was encapsulated within the PVA fibers to ensure sustained release. The incorporation of SDF-1 into the PVA fibers provided significant immunomodulatory benefits. The SDF-1-loaded membranes reduced inflammation by modulating immune responses and promoting cell proliferation, migration, and angiogenesis. This immunomodulation is crucial for enhancing healing, particularly in chronic wounds of diabetic patients [177]. Another study was about the use of co-axial electrospinning to generate core/shell poly(lactic-co-glycolic acid) (PLGA)/gelatin fibers. These fibers were designed to deliver bioactive molecules, including SDF-1α, VEGF-binding peptide (BP), and liraglutide (LG), to enhance wound healing. This scaffold incorporates (i) SDF-1α for recruiting endogenous stem and (ii) progenitor cells and angiogenic molecules (BP and LG) to promote tissue repair synergistically. This combination facilitated immune modulation by reducing inflammation and promoting the migration and proliferation of endothelial cells, thereby enhancing neovascularization. Histologically, the treated wounds showed better re-epithelialization, collagen deposition, and skin appendage (hair follicles and glands) regeneration, with reduced inflammation compared to controls [182].

The use of a tailored fibrin hydrogel scaffold with low stiffness was shown to positively influence the healing process of mouse skin wounds through decreasing neutrophil and increasing non-classical Ly6C^low^ monocyte and resolutive macrophage (CD206+ and CX3CR1+) populations, at day 3 after injury. Fibrin hydrogel at 3.5mg/mL fibrinogen reduced the expression of pro-inflammatory cytokines and increased IL-10 levels. Frequencies of dermal endothelial cells, fibroblasts, and keratinocytes increased, and fibrin hydrogel enhanced keratinocyte migration [187].

## 9. Why SDF-1/CXCL12 May Stand Out for Wound Healing

Above we exemplify critical mechanisms of skin wound healing and extend detailed insights in cell biological and medical aspects of normal [27] and abnormal [28] wound healing. In Figure 1 and Table 1, we complement these insights with an overview of critical molecules in specific phases of skin wound healing. The sequential and phase-specific appearance of predominant leukocyte types and their dissipation in later phases in wounded and healing skin are orchestrated by chemokines with specific signatures. In humans, IL-8/CXCL8 and related CXC chemokines first recruit neutrophils, and thereafter monocytes are attracted by CC-chemokines (e.g., MCP-1/CCL2, MCP-2/CCL8 and MCP-3/CCL7), whereas other mononuclear leukocytes that are less mobile (lymphocytes and natural killer cells) come in last, possibly by their inertia (having less propulsion power because of less motor molecules) but also because the chemotactic profiles are altered gradually by resident tissue cells and recruited leukocytes. With the focus on the chemokine system and specifically on SDF-1/CXCL12 in wound healing, a number of points need attention. From the genetic spectrum and the resulting new nomenclature of chemokines, it emerges that the chemokine system is complex, with 44 ligands and 22 receptors in the human species [188]. In other words, a detailed understanding of chemokine biology is needed if one chooses one specific factor for clinical use. Second, within the order of vertebrates, zebrafish have 100 chemokine genes, whereas the mouse has only 38 genes [187]. Hence, due to considerable differences in circulating leukocyte numbers and functionalities across species (see below), biological data, including observations of chemokines in animal models of wound healing, need to be interpreted with caution and may only be used as preamble for good clinical studies. Well-executed clinical studies will remain essential in order to improve the burden for difficult-to-heal skin wounds.

The skin has multiple layers and cell types, each cell type being responsive to rather specific growth factors for their proliferation. Instead of reviewing details of EGF for keratinocytes, VEGF and placental growth factor (PlGF) for endothelial cells, fibrobast growth factors (FGFs) and TGFs for (myo/)fibroblasts, we here address the question of why all these growth factors failed clinically for skin wound healing, whereas PDGF became successful. The answer may be related to the origin of PDGF. Indeed, as its name indicates, PDGF is abundantly provided by degranulating platelets within minutes after skin wounding, and it is a universal growth factor for all named skin tissue cell types: keratinocytes, endothelial cells and fibrobasts (not to name melanocytes, adipocytes, mast cells, etc.). Of notice, PDGF is also the most important growth factor in plasma/serum and, when using autologous plasma-derived fibrin clots for tissue regeneration, PDGF is included as a major growth factor.

In analogy with the broad cell specificity of PDFG stated above, the question about which chemokine to use for regeneration of wounded skin may be solved in at least two ways: (i) looking for a chemokine produced by platelets, and/or (ii) defining a chemokine with broad cell specificity. When focusing on abundantly released platelet-derived chemokines, platelet factor-4 (PF-4/CXCL4), including its variant PF-4var/CXCL4L1, stands out as the first purified chemokine of which the complete protein sequence (1977) was known long before its cDNA cloning was accomplished as the 4th historical CXC chemokine a decade later in 1987 [189]. Like PDGF, PF-4/CXCL4 is also released from platelet α-granules and executes important negative feedback control mechanisms, such as inhibition of hematopoiesis and angiogenesis. A gene variant of PF-4/CXCL4 was identified, and it was established that the encoded protein PF-4var/CXCL4L1 is at least five times more potent in inhibition of angiogenesis [190]. Detailed comparisons of PF-4/CXCL4 and PF-4var/CXCL4L1 were comprehensibly reviewed [191].

**Figure 3:**
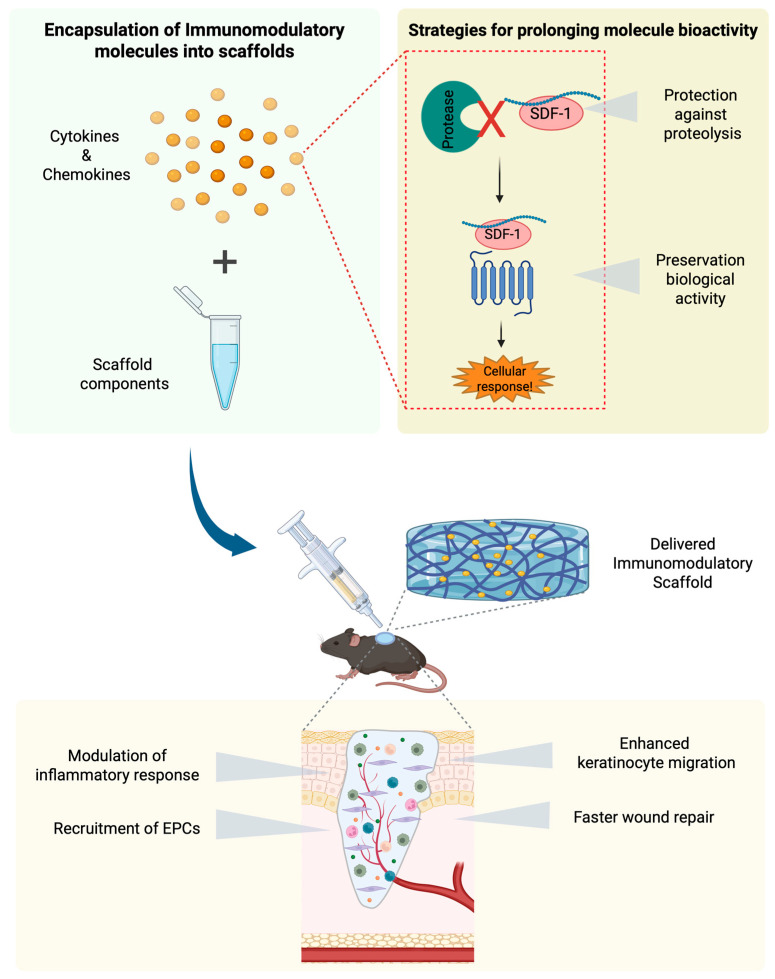
Cytokines and chemokines for skin wound healing. Immunomodulatory molecules, such as cytokines and chemokines, may be incorporated into scaffolds for delivery to promote skin wound healing. SDF-1 is highlighted as an example with beneficial effects on wound repair. One strategy to prolong SDF-1 bioactivity is to protect it from proteolytic degradation, ensuring sustained activity at the wound site. The therapeutic delivery of SDF-1 aims to modulate the inflammatory response, recruit endothelial progenitor cells (EPCs), enhance keratinocyte migration, and accelerate wound healing. This and other combination approaches have been experimentally tested in preclinical animal models, and these investigations form a stimulus for better and more pharmacological clinical studies. Figure created in BioRender.

CXCL12 stands out as a druggable chemokine for skin wound healing because (i) it is not provided in large quantities by platelets and other blood-derived elements in the various phases of skin wound healing; (ii) its physiological function as a leukocyte chemokine is directed towards calling CD34-positive hematopoietic cells back to the bone marrow, hence its plasma concentrations are regulated to remain low; and (iii) progenitor cells for almost all (skin) tissue cells, including keratinocytes, endothelial cells and fibroblasts as major examples, carry CXCR4 as the cognate receptor of SDF-1/CXCL12. Therefore, when exogenous SDF-1/CXCL12 is locally applied to wounds as a pharmacological agent and in ways that preserve its bioactivities, it will exert potent chemotactic activity for all necessary cells for a normal healing process while preserving all control mechanisms and avoiding systemic side-effects (Figure 3).

## 10. SDF-1/CXCL12 Beyond Wound Healing

A primary function of the SDF-1-CXCR4 axis operates in the stroma of the bone marrow and is capable—during acute infections and inflammations when neutrophils move massively from the bone marrow to the inflammatory sites via the circulation—to call back “escaped” hematopoietic progenitor cells to the bone marrow niches. Indeed, early studies demonstrated that stem cell engraftment into the bone marrow needs SDF-1 [192], and this implies that, for the chemotactic activity of SDF-1, its normal levels need to be and remain extremely low in the blood circulation. During infections and inflammatory reactions, the temporarily raised SDF-1 plasma concentrations may easily be lowered by the “killing” of bioactive SDF-1/CXCL12 by neutrophil proteinases [108] (vide supra). Circulating pro-inflammatory cytokines associated with inflammatory reactions, including colony stimulating factors, such as G-CSF, result in a feedback decrease in SDF-1 levels in the bone marrow [19]. The proven observation of low circulating levels of SDF-1/CXCL12 [193] has been cleverly exploited in medicine for mobilization of hematopoietic stem cells into the circulation and prohibition of their return into the bone marrow compartment. In this way, CXCR4-positive hematopoietic cells may be easily collected by a simple venous blood tap. This procedure nowadays replaces painful bone marrow punctures for transplantation purposes. Hematopoietic stem cell mobilization may thus be obtained by intravenous injection of SDF-1/CXCL12 [193] or by antagonizing the SDF-1-receptor CXCR4 with the use of the small molecular weight antagonist AMD3100, named Plerixafor [reviewed by [194]]. This drug was originally developed against AIDS when it was established that human immunodeficiency virus-1 (HIV-1) may use CXCR4 as an entry co-receptor to infect T lymphocytes [23,24,195].

Insights into a locally operating SDF-1-CXCR4 axis in various tissues and the knowledge that all kinds of stem cells show plasticity have been exploited in all possible “directions”. For instance, locally recruited stem cells into specific tissues may be redirected to the bone marrow for hematopoietic purposes. SDF-1 exerts additional functions within the bone marrow in maintaining hematopoietic stem cells and lymphoid precursors in specialized niches, either perivascular or endosteal, as evidenced by experiments in which expression of SDF-1 was deleted in specific cell types [196,197]. Later, a perisinusoidal niche was described for extramedullary hematopoiesis in the spleen where stromal cells were the main producer cell type of SDF-1 [198].

As another application, bone marrow-derived stem cells may be recruited to the periphery for regenerative medicine purposes. Many examples of in vitro or in vivo studies have meanwhile been published to exemplify the actions of SDF-1/CXCL12 for regenerative medicine. These include studies on muscle tissue [199,200,201], intestinal epithelial regeneration [202], burned skin [203,204], airway [205] and bone repair [206,207,208], neuronal guidance [209] and neurite [210] and motor neuron axonal outgrowth [211], intervertebral disc regeneration [212], liver regeneration [213], cartilage [214] and meniscal defect repair [215], corneal epithelial regeneration [216], regeneration of smooth and striated muscles in anal sphincter defects [217], attempts towards in vivo repair of neuronal tissue or cells [218] and spinal cord injuries [219]. In most of these studies, the origin of the SDF-1/CXCL12 ligand is from mesenchymal cells, not only bone marrow stromal cells but also interstitial tissue or organ fibroblasts and even mesenchymal stem cells. As stated above, platelets are poor producers of SDF-1/CXCL12, yet when abundantly present and activated, as in acute skin wound healing, may assist in the healing process thanks to SDF-1 [220]. Local production of SDF-1/CXCL12 and GAG-binding of this ligand makes all types of progenitor cells, including angioblasts, bone marrow-derived, and other types of stem cells carrying the cognate receptor CXC4 on their membranes are recruited and locally stimulated into proliferation and differentiation. Within the central nervous system, a notable example for future regenerative medicine applications is the differentiation of oligodendrocytes progenitor cells leading to remyelination [221].

In addition, this SDF-1-CXCR4 axis is induced by hypoxia conditions and is thus also naturally active in all instances of tissue ischemia [115,222]. This element has been extensively studied for myocardial infarction and ischemic cardiomyopathy [223,224,225,226,227]. A critical note relates to details about in vivo studies. On the one hand, the use of gene-deleted mice, e.g., *Cxcr4*-/- mice, led to the finding that not all SDF-1/CXCR4-mediated processes are beneficial for tissue regeneration in an animal model of myocardial infarction [228]. On the other hand, in vivo studies for fin regeneration in zebrafish shows that even vasculogenesis of new arteries is dependent on a functional SDF-1-CXCR4 system [229]. Together, the above examples illustrate that SDF-1/CXCL12 in various formulations may be used for acute and chronic skin wound repair, but that similar and new delivery systems also have applications in various medical disciplines beyond dermatology.

## 11. Conclusions and Perspectives

Historically, SDF-1/CXCL12 was discovered by chance, and studies of the SDF-1/CXCL12-CXCR4 axis were skewed towards investigations of cancer cell biology and the roles played by SDF-1 and stem cells in the processes of invasion and metastasis of cancer cells [230]. In this review, the emphasis has been on (i) the role of this axis in wound healing and normal organogenesis and (ii) how insights from the biology of SDF-1 and its pharmacological conditioning may be used as an example for applications of other cytokines, such as EGF and VEGF, in regenerative medicine. About a decade ago, SDF-1/CXCL12 was already suggested as a key reagent for therapeutic angiogenesis, wound healing and tissue regeneration [231,232]; however, only a small number of convincing studies were reported. From the present review it becomes clear that, meanwhile, a number of key points are worthwhile to remember and to apply (Box 1).

Box 1 **Key points to remember.**

- Treatments of acute and chronic skin wounds need to be based on etiology, interference with causal factors and solid preclinical research. Although cytokines and growth factors are important regulators of skin wound healing, only PDGF is FDA-approved so far.
- Because skin and appendages differ considerably across species and numbers of immune cells and levels and balances of cytokines and growth factors greatly vary, even when comparing mammalian species, promising preclinical tests in mice and pigs may still yield different outcomes in humans. Carefully executed clinical studies will remain essential for skin wound healing and for regenerative medicine research.
- Stromal-derived factor-1, alias CXCL12, is a druggable protein with multiple beneficial functions for skin wound repair, including recruitment of hematogenic progenitor cells for all skin cell types and for endothelial cells in the granulation phase. Insights about its biology and various formulations may be useful for the applications of other biotechnological agents in regenerative medicine.
- SDF-1/CXCL12 bioavailability for topical application in skin wounds may be enhanced with the use of prolonged delivery systems and scaffolds, including natural proteins and bio-synthetic polymers. Ideally, SDF-1/CXCL12 needs also to be protected against proteolytic degradation to prolong its bioavailability.

**Figure 4:**
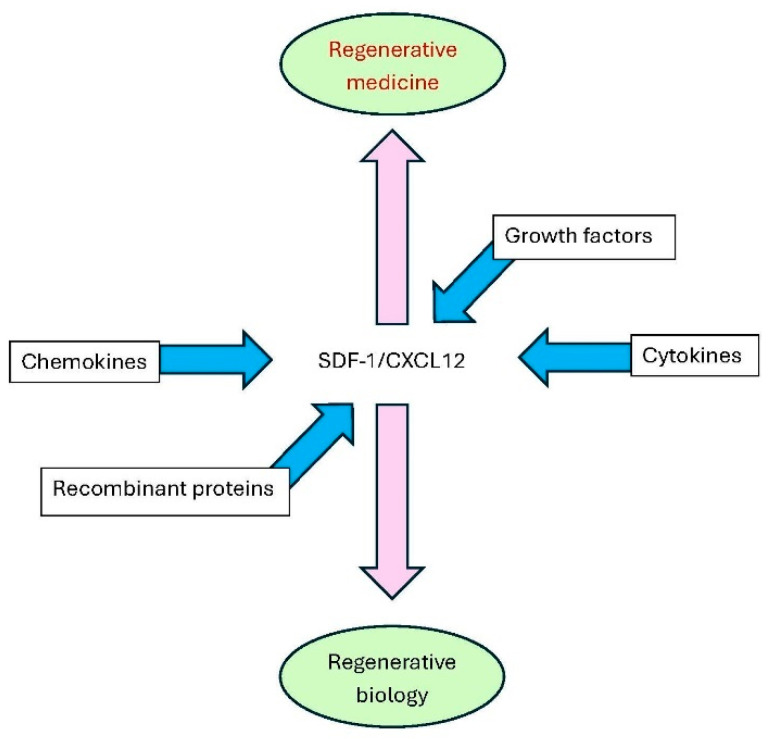
SDF-1/CXCL12 is central at the crossroads between regenerative biology and medicine. SDF-1/CXCL12 was characterized as belonging to a small protein family having a CXC sequon and later recognized to encode cytokines with chemotactic activity and dubbed chemokines. By acting mainly through the G protein-coupled receptor CXCR4, it also mediates hematopoietic functions and assists in VEGF-induced angiogenesis by retention of CXCR4-positive bone marrow-derived precursor cells [234]. The beneficial effects on precursor cells from different lineages in normal organogenesis and tissue regeneration stimulated studies of SDF-1 for wound healing. These studies become now preambles for future investigations of SDF-1, which range from regenerative biology to regenerative medicine. Recombinant SDF-1/CXCL12 has been used with some success in various pharmacological formulations for wound healing. The low circulating and local tissue concentrations of SDF-1 and the fact that it binds to glycosaminoglycans enable in situ use for regenerative medicine applications and raise hope for applications beyond the discipline of dermatology.

In view of the increasing need for better therapies for acute and chronic skin wounds, the difficulties in performing in vitro basic research based on the complex nature of skin and the lack of appropriate models with all tissue elements, we here complement detailed information on major cellular players in the various phases of normal wound healing with new insights into how SDF-1/CXCL12 and other cytokines and growth factors in combination with specific pharmacological delivery agents may change future treatments of difficult-to-heal or chronic skin wounds. Logically and aside from SDF-1/CXCL12, growth factors for specific skin cells, e.g., EGF for keratinocytes, FGFs and TGFs for dermal fibroblasts/myofibroblasts, VEGF and PlGF for endothelial cells, may also be tuned and further conditioned for skin regeneration. However, so far only PDGF with growth potential for all the named cell types is in clinical use [233]. Aside from cell growth, local chemotaxis of progenitor cells for all tissue layers may add efficiency in wound healing processes. Although much research is still needed to discover causal factors of impaired wound healing and (combinations of) molecules that drive skin tissue regeneration, SDF-1/CXCL12 is gradually moving towards the pole position in biotechnology as a key regenerative agent for skin and other tissues and organ systems and is getting a place at the center between regenerative biology and medicine (Figure 4). This implies that many efforts are now focused on improving the continuous delivery, the stability against proteolytic degradation and optimized formulations of SDF-1/CXCL12. Research with newly biofabricated scaffolds for delivery of other cytokines and growth factors for improved regenerative medicine also deserves renewed interest. It is everyone’s hope that the outlined insights will yield new products and approaches based on solid research in molecular biology and biochemistry.

## Figures and Tables

**Table 1:** Wound healing phases, major cell actions and molecules.

Wound Healing Phase (Time Interval)	Major Cell Actions	Major Molecules, Cytokines, Growth Factors	References
**Hemostasis** (seconds to minutes)	Cellular and endothelial damage Platelet aggregation	Vasoconstrictors von Willebrand factor (vWF) Growth factors (PDGF, TGF-β, EGF, VEGF, IGF-1) Matrix metalloproteinases (MMPs) Chemokines (e.g., SDF-1/CXCL12, CXCL4) Coagulation factors (Factor Xa, Thrombin, Fibrinogen, Fibrin) Complement factors	[128,129,130,131,132,133]
**Inflammation** (days)	Neutrophil recruitment (d2) Monocyte recruitment (d3) Macrophage activation and polarization (d4) Lymphocytes, NK cells (d5) Clearance of cell debris	DAMPs, PAMPs Hydrogen peroxide Lipid mediators (mainly LTB4) Chemokines (CXCL8, CCL2, …) Reactive oxygen species (ROS) Pro-inflammatory cytokines (IL-1β, TNF-α, IL-6)	[15,16,17,27,28,134,135,136,137,138,139,140,141]
**Proliferation &** **Granulation** (weeks)	Granulation tissue formation Angiogenesis Lymphangiogenesis Endothelial progenitor cell recruitment Fibroblast proliferation Myofibroblast differentiation	Growth factors (VEGF, FGF, PDGF, TGF-β) ECM (Collagen type III, Fibronectin, Proteoglycans) α-smooth muscle actin (α-SMA) Matrix metalloproteinases (MMPs)	[142,143,144,145,146,147,148]
**Remodeling** (months to years)	Collagen remodeling by fibroblasts Scar tissue formation Vascular regression	Matrix metalloproteinases (MMPs) Tissue inhibitors of metalloproteinases (TIMPs) Collagen type I	[28,148,149,150]

**Table 2:** Methods of immunomodulator incorporation, controlled release mechanisms and bioactivity testing.

Immunomodulator	Scaffold	Incorporation Method	Controlled Release Mechanism	Bioactivity-Test	Ref.
SDF-1/CXCL12	PVA/PCL electrospun membrane	Encapsulation	Burst release followed by diffusion because of polymer chain relaxation	Cell migration	[177]
SDF-1/CXCL12	PLGA/gelatin electrospun membrane	Encapsulation	N/A	N/A	[182]
SDF-1/CXCL12	Gelatin microgel	Freeze drying	Degradation-based	Cell migration	[185]
Hydrogel: physico-chemical properties	Fibrin hydrogel	Not applicable	N/A	Reduction in pro-inflammatory cytokines and increase in IL-10 levels	[187]
SDF-1/CXCL12	Fibrin hydrogel	Binding to COAM (GAG mimic molecule) and encapsulation	Burst release followed by diffusion	Biochemical analysis of chemokine integrity and cell migration	[108]
IL-8	PLGA nanoparticles	Encapsulation	N/A	Cell proliferation and migration	[181]
Exosomes from MSCs	Hyaluronic acid, gelatin, and chitosan hydrogel	Encapsulation	Diffusion	Cell proliferation and reduction in pro-inflammatory cytokines	[184]

N/A means not analysed.

## Data Availability

The data presented in this study are openly available in [Lirias] [https://kuleuven.limo.libis.be/discovery/search?vid=32KUL_KUL:Lirias&sortby=rank&fromLogin=true] accessed on 11 January 2026.
